# PMP2 Enhances Schwann Cell Metabolism and Promotes Myelination

**DOI:** 10.1111/jnc.70265

**Published:** 2025-10

**Authors:** Gustavo Della-Flora Nunes, Jiayue Hong, Rebekah Garfolo, Acheta Jenica, Amanda S. Mondschein, Olivia M. Harris, Khushi Panchal, Frances L. Jourd’heuil, David Jourd’heuil, Yannick Poitelon, Sophie Belin

**Affiliations:** 1 Department of Cell and Developmental Biology, University of Colorado School of Medicine, Aurora, Colorado, USA; 2 Department of Neuroscience and Experimental Therapeutics, Albany Medical College, Albany, New York, USA; 3 Department of Molecular and Cellular Physiology, Albany Medical College, Albany, New York, USA

**Keywords:** ATP, PMP2, Schwann cell, seahorse

## Abstract

Myelinating Schwann cells depend on precise metabolic regulation to support axonal function and maintain peripheral nerve integrity. Peripheral Myelin Protein 2 (PMP2), a fatty acid-binding protein enriched in myelinating Schwann cells, has been implicated in lipid metabolism and mitochondrial energy production. Here, we examine the role of PMP2 in regulating Schwann cell bioenergetics and myelination. Using both immortalized and primary Schwann cells, we show that PMP2 overexpression enhances mitochondrial ATP production. We also reveal that PMP2 alters metabolic dependencies during high metabolic demand, reducing Schwann cell reliance on glutamine while promoting greater metabolic adaptability under substrate restriction. Finally, PMP2 overexpression significantly increases myelination in vitro, indicating that PMP2-driven metabolic modulation supports the energetic demands of myelination. These findings position PMP2 as a key regulator of Schwann cell metabolism and a potential therapeutic target for demyelinating neuropathies.

## 1 | Introduction

The peripheral nervous system (PNS) relies on the intricate interplay between neurons and glial cells to maintain its functionality. Among these glial cells, Schwann cells play a pivotal role by forming the myelin sheath that insulates axons and by providing critical metabolic support to neurons (Bosch-Queralt et al. 2023). In doing so, Schwann cells not only ensure rapid saltatory conduction but also contribute to the health and survival of peripheral axons. Myelination is an energy-intensive process. To generate the extensive multilamellar myelin sheath, Schwann cells must synthesize large quantities of lipids and proteins, requiring a robust metabolic infrastructure to meet high biosynthetic and adenosine triphosphate (ATP) demands (Poitelon et al. 2020; Ravera et al. 2020; Silva et al. 2025). Mitochondria are central to this process, supporting lipid synthesis and oxidative phosphorylation for sustained ATP production. Mitochondrial dysfunction in Schwann cells is increasingly recognized as a contributing factor in various peripheral neuropathies and demyelinating diseases (Ino and Iino 2017). Interestingly, recent studies suggest that while mitochondrial metabolism is important, it may not be strictly essential for myelination itself. In the central nervous system (CNS), oligodendrocytes can maintain myelination even when oxidative phosphorylation is compromised, relying instead on glycolysis and exporting lactate to axons as an energy source (Funfschilling et al. 2012). Similarly, in the PNS, Schwann cell-specific knockout of pyruvate dehydrogenase (PDHA1), an enzyme required to feed glycolysis-derived pyruvate to the tricarboxylic acid cycle in mitochondria, does not impair myelination or long-term maintenance of the myelin sheath (Della-Flora Nunes et al. 2017). These observations raise the possibility that glycolytic pathways and lipid-derived metabolites might compensate for impaired mitochondrial respiration. However, Schwann cell-specific ablation of ATP citrate lyase (ACLY), which provides cytosolic acetyl-CoA for de novo fatty acid synthesis, impairs myelin maintenance but not myelination by limiting membrane lipid availability, even when mitochondrial oxidative capacity remains intact (Schneider et al. 2025). Together, these findings suggest that while Schwann cells possess metabolic flexibility to maintain energy production, the synthesis of lipids, rather than ATP generation, is the critical metabolic bottleneck for proper myelination.

Beyond myelination, Schwann cells actively support axonal metabolism through a process known as metabolic coupling. Schwann cells supply axons with energy substrates such as lactate and pyruvate, primarily derived from glycolysis. These metabolic exchanges become particularly important under conditions of high activity or injury, when axonal energy demand increases (Babetto et al. 2020). Disruption of this support system has pathological consequences. For instance, the deletion of lactate dehydrogenase, the enzyme responsible for converting pyruvate to lactate and vice versa, in Schwann cells leads to progressive axonal degeneration (Bloom et al. 2022). Similarly, the deletion of pyruvate kinase M2, which supports the conversion of phosphoenolpyruvate to pyruvate with the production of ATP in the final reaction of glycolysis, affects axon physiology, without affecting myelination (Deck et al. 2022). Finally, conditional deletion of mitochondrial transcription factor A (Tfam) in Schwann cells disrupts lipid homeostasis, shifting metabolism toward fatty acid oxidation and leading to progressive demyelination and axonal loss (Viader et al. 2013; Viader et al. 2011). These findings highlight Schwann cells not just as passive supporters, but as active metabolic partners essential for both axonal maintenance and regeneration.

Interestingly, myelin lipids themselves may serve as an energetic reservoir, contributing to axonal support during metabolic stress (Morelli et al. 2011; Asadollahi et al. 2024). Recent studies have highlighted the intercellular crosstalk between adipocytes and Schwann cells. Following injury, the adipokine leptin, produced by adipocytes, acts as a signal detected by Schwann cells and triggers a metabolic switch from lipid storage to energy production. This may involve redirecting fatty acids from myelin to fuel mitochondria and boost energy production, thereby helping nerve repair (Sundaram et al. 2023).

The fatty acid binding proteins (FABPs) are fatty acid chaperones that may facilitate the redistribution of fatty acids within subcellular compartments in Schwann cells. FABP8, also known as PMP2, is predominantly expressed in myelinating Schwann cells and, following a mosaic pattern of expression, is preferentially expressed by Schwann cells myelinating large-caliber motor axons (Kozlowski et al. 2025; Yim et al. 2022). PMP2 is known to bind and transport fatty acids to membranes and to play a role in lipid homeostasis within peripheral nerves (Zenker et al. 2014), but its exact function in Schwann cells’ metabolic function is still unclear. In PMP2 knockout mice, transient changes in lipid composition and nerve conduction velocity were observed, suggesting PMP2’s importance for maintaining lipid homeostasis to preserve nerve function during development (Stettner et al. 2018; Zenker et al. 2014). High expression of axonal Neuregulin 1 type III (NRG1t3) is known to increase myelin thickness during development (Michailov et al. 2004; Taveggia et al. 2005) and during remyelination (Stassart et al. 2013). We found in prior studies that high expression of NRG1t3 correlated with increased expression of PMP2 in sciatic nerves during development (Scapin et al. 2019; Belin et al. 2019) and following nerve crush injury (Hong et al. 2024). Furthermore, during remyelination increased PMP2 expression is associated with enhanced fatty acid uptake and increased mitochondrial ATP production in mouse sciatic nerves, linking PMP2 to broader metabolic functions (Hong et al. 2024).

Together, these studies underscore the dual reliance of Schwann cells on aerobic and anaerobic pathways to sustain their functions. PMP2 may play a central role in this metabolic flexibility by linking lipid handling to mitochondrial ATP production and fatty acid β-oxidation. In this study, we investigate the role of PMP2 in regulating Schwann cell myelination and energetic metabolism.

## 2 | Methods

### 2.1 | Animals

All animal procedures were approved by the Albany Medical College Institutional Animal Care and Use Committee (IACUC protocol no. 23-08001). All procedures conformed to ARRIVE guidelines and the principles of the Basel Declaration. The choice of different ages and species was driven by experimental needs as follows. Both male and female embryos/pups were used. Postnatal Day 5 (P5) or Day 40 (P40) mice were used for primary Schwann cell isolation and were used to compare metabolic states across maturation stages. Three mice at each age were used. C57BL6/J mice were purchased from the Jackson Laboratory. P2 rats were used for Schwann cell isolation to produce robust primary cultures, capable of efficient lentiviral transduction and in vitro myelination. Twelve rats were used. Embryonic Day 15.5 (E15.5) rat embryos were used for dorsal root ganglion (DRG) neuron isolation and were used in coculture with rat Schwann cells to assess myelination. Three dams and about 24 embryos were used. Sprague–Dawley rats were purchased from Taconic.

### 2.2 | Primary Cell Isolation

For mouse primary Schwann cells, P5 mice were euthanized by decapitation, and P40 mice were euthanized using carbon dioxide gas and prepared similarly to (Della-Flora Nunes, Wilson, Marziali, et al. 2021). Briefly, sciatic nerves were dissected at P5 or P40, stripped of epineurium and other contaminant tissues (connective tissues, adipocytes), pooled, and kept in culture for 7 days to allow for Schwann cell dedifferentiation and their isolation. Cells were maintained in media containing high glucose Dulbecco’s Modified Eagle Medium (DMEM) (4.5 g/L glucose) supplemented with 10% fetal bovine serum, 2 mM L-glutamine, 100 U/mL penicillin, 100 μg/mL streptomycin, 10 ng/mL Nrg1 (human NRG1-β1 extracellular domain, R&D Systems Cat. No. 377-HB), and 2 μM forskolin. After 7 days, cells were dissociated enzymatically using a mixture of 2.5 mg/mL of dispase II (Sigma-Aldrich) and 130 U/mL of type I collagenase (Worthington Biochemical Corporation) for 3 h at 37°C, mechanically dissociated using fire-polished glass pipettes, and seeded on one 35 mm dish coated with laminin (Sigma-Aldrich). Mouse Schwann cells were maintained for up to a week in high glucose DMEM (4.5 g/L glucose) supplemented with 2 mM L-glutamine, 100 U/mL penicillin, 100 μg/mL streptomycin, 2 ng/mL Nrg1, and N2 supplement (Thermo Fisher Scientific).

For primary rat Schwann cells, P2 rats were euthanized by decapitation. Sciatic nerves were pooled, and Schwann cells were isolated as described (Weaver et al. 2025) and were not passed more than six times. Cells were maintained in media containing high glucose DMEM (4.5 g/L glucose) supplemented with 10% fetal bovine serum, 2 mM L-glutamine, 100 U/mL penicillin, 100 μg/mL streptomycin, 2 ng/mL Nrg1, and 2 μM forskolin.

### 2.3 | DRG Neuron Isolation and Co-Culture With Schwann Cells

DRG neurons were isolated from E15.5 rat embryos, pooled, and prepared as described (Catignas et al. 2021; Wilson et al. 2024). Briefly, dams were culled using carbon dioxide gas, and embryos were culled by decapitation. DRGs were dissected, cleaned, pooled, and dissociated enzymatically (0.25% trypsin-ethylenediaminetetraacetic acid for 25 min at 37°C). Cells were triturated and plated onto 2 cm^2^ wells with poly-L-lysine/laminin-coated coverslips in Neurobasal medium supplemented with B-27 (Gibco), 2 mM glutamine, 50 ng/mL Nerve Growth Factor, and antibiotics. Cultures were maintained for 7 days to allow axon outgrowth. DRG rat neurons were purified by complementing the Neurobasal medium with antimitotic agents Fluoro-2-deoxyuridine (Sigma Cat. No. F-0503) and uridine (Sigma Cat. No. U-3003) to remove non-neuronal cells. For myelination assays, 100,000 Schwann cells (GFP- or PMP2-GFP-infected) were seeded on top of DRG neuron cultures and maintained in myelination media (DMEM + 10% FBS + 50 μg/mL ascorbic acid). Myelination was assessed after 14 days with immunostaining.

### 2.4 | S16 Schwann Cell Culture

S16 rat Schwann cells (ATCC, CRL-2941; RRID:CVCL_B072). S16 are not listed as a commonly misidentified cell line by the International Cell Line Authentication Committee. S16 were cultured in high-glucose DMEM (4.5 g/L) and 5% fetal bovine serum. We selected S16 cells because they maintain robust expression of myelin genes (e.g., *Pmp22*, *Mpz*, *Pmp2*) are well characterized. Authentication was confirmed by epithelial-like morphology and myelin marker expression within the last 12 months (Figure 2A). S16 Schwann cells were passed a maximum of 12 times.

### 2.5 | Primary Schwann Cell and S16 Lentiviral Infection

Lentivirus virions for Green Fluorescent Protein (GFP) (Origene, Cat. No. PS100071V) and PMP2-GFP (Origene, Cat. No. RR210206L2V) were purchased pre-packaged from Origene. For infection, Schwann cells (primary or S16) were plated at 1 × 10^5^ cells per 2 cm^2^ well. A multiplicity of infection of 10:1 was used. Cells were incubated with virions in growth media supplemented with 8 μg/mL polybrene for 6 h. After 72 h, cells were either fixed for imaging or used for metabolic assays. Infection efficiency was confirmed by GFP fluorescence and ranged from 60% to 80%.

### 2.6 | Metabolic Flux Analysis

**FIGURE 1 |:**
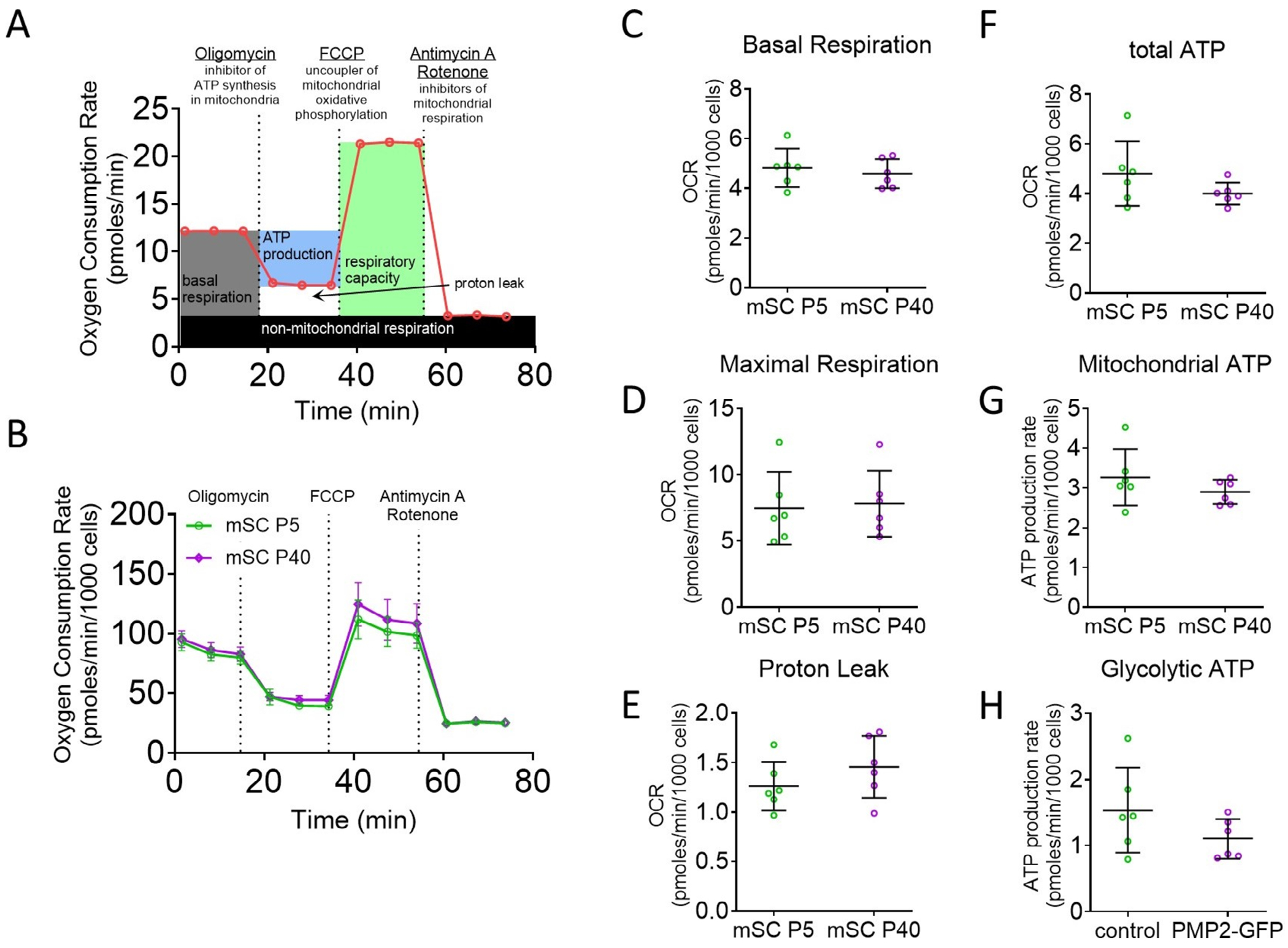
Isolated Schwann cells at different developmental ages show similar metabolic responses. (A) Oxygen consumption rate in mouse Schwann cells (mSC) isolated at postnatal Day 5 (P5) and P40. (B) Schematic representation of the mitochondrial stress test. (C–H) Basal respiration (C), maximal respiration (D), proton leak (E), total ATP production (F), mitochondrial ATP (G), and glycolytic ATP production (H) were extrapolated from the OCR and ECAR measurements in P5 and P40 mSCs. Error bars represent SD, *n* = 6 wells from 2 distinct experiments, and each point on the graph represents a different *n*.

Cellular bioenergetics analysis was performed using a Seahorse XFp instrument and the mitochondrial stress test (Agilent), following the manufacturer’s instructions to dynamically measure the extracellular acidification rate (ECAR), a surrogate marker of glycolysis, and the oxygen consumption rate (OCR), a surrogate for basal oxygen consumption. Schwann cells were plated on poly-L-lysine coated wells of the XFp plate in a number that resulted in a confluent layer of cells by the time of the analysis. During the mitochondrial stress test, Schwann cells were kept in non-buffered XF DMEM medium pH 7.4 supplemented with 1 mM pyruvate, 2 mM glutamine, and 10 mM glucose. For the assay, 1.5 μM oligomycin was used to inhibit mitochondrial ATP synthesis, 2 μM carbonyl cyanide-p-trifluoromethoxyphenylhydrazone (FCCP) was used to stimulate maximal mitochondrial respiration, and 0.5 μM rotenone and 0.5 μM antimycin A were used to completely block mitochondrial respiration (Figure 1A). At the end of the assays, cell nuclei were stained with Hoechst 33342 (Thermo Fisher Scientific), and a Biotek Cytation 5 plate reader was utilized to obtain an automated cell count. Results were normalized by cell number. Formulas to calculate the extrapolations from H^+^ production and O_2_ consumption are as follows: Basal Respiration (minimal basal OCR—minimal Antimycin A/Rotenone OCR), Maximal Respiration (maximal FCCP OCR—minimal Antimycin A/Rotenone OCR), ATP Production (minimal basal OCR—minimal Oligomycin OCR), Proton leak (minimal Oligomycin OCR—minimal Antimycin A/Rotenone OCR), Mitochondrial ATP Production (ATP Production × 2 × P/O ratio [number of ATP molecules generated per atom of oxygen]), Glycolytic ATP Production (maximal basal ECAR × volume of microchamber × Kvol × Buffer Factor—ATP Production × CO_2_ contribution Factor (CCF)). Here, P/O was set at 2.75, volume of microchamber at 2.28, Kvol to 1.6, Buffer Factor to 2.5, and CCF at 0.61.

For the substrate oxidation stress test, Schwann cells were treated similarly as above, except for the following modifications. During the assay substrate oxidation stress test, the media was supplemented with either 5 mM PYR (Pyruvate solution, Agilent Cat. No. 103578-100) to test glycolytic dependency/capacity, 20 mM GLN (Glutamine solution, Agilent Cat. No. 103579-100) to test glutaminolysis dependency/capacity, or 100 μM oleic acid (OA) (Sigma-Aldrich Cat. No. O1008) to test fatty acid dependency/capacity/capacity. For the assay, 2 μM UK5099 was used to inhibit the oxidation of glucose and/or pyruvate, 3 μM Bis-2-(5-phenylacetamido-1,3,4-thiadiazol-2-yl)ethyl sulfide (BPTES) was used to inhibit the oxidation of glutamine, and 4 μM etomoxir was used to inhibit the oxidation of long-chain fatty acids (Figure 4A). Formulas to calculate the extrapolations from H^+^ production and O_2_ consumption are as follows: Acute Response (minimal inhibitor OCR—minimal Antimycin A/Rotenone OCR).

To account for variations in cell seeding and ensure accurate metabolic measurements, all Seahorse assay data were normalized to cell number per well. Immediately following each metabolic assay, cells were stained with Hoechst 33342 (5 μg/mL in phosphate buffered saline (PBS)) for 15 min at 37°C. Plates were imaged using a BioTek Cytation 5 imaging plate reader, and total nuclear counts per well were quantified using Gen5 software (BioTek) with standardized thresholding parameters. Oxygen consumption and extracellular acidification rates (OCR and ECAR) were normalized to the number of nuclei per well and reported as pmol/min/cell. This approach allows direct comparison of metabolic activity between groups independent of cell density variability.

### 2.7 | Immunocytochemistry

Cells or co-cultures were fixed in 4% paraformaldehyde for 15 min at room temperature, permeabilized with 0.2% Triton X-100 in phosphate-buffered saline for 10 min, and blocked in 5% normal goat serum for 1 h as described (Della-Flora Nunes, Wilson, Hurley, et al. 2021). Primary antibodies were incubated overnight at 4°C in blocking solution. Antibodies included anti-Myelin Basic Protein (MBP) (Biolegend, Cat. No. 808401, RRID: AB_10120129), anti-Neurofilament M Protein (NFM) (Biolegend, Cat. No. 822701, RRID:AB_663194), all at 1:500. After PBS washes, Alexa Fluor-conjugated secondary antibodies (1:1000, Thermo) were applied for 1 h at room temperature. Nuclei were counterstained with 4′,6-diamidino-2-phenylindole (DAPI). Coverslips were mounted using ProLong Glass and imaged on a Zeiss Axio Imager A2 epifluorescence microscope. For quantification of myelination, two random 10× fields per coverslip were analyzed using ImageJ to count and measure MBP-positive segments, as described in (Acheta et al. 2022).

### 2.8 | Western Blotting

Cells were lysed in radioimmunoprecipitation assay buffer supplemented with protease and phosphatase inhibitors. Protein concentration was measured using the Bicinchoninic Acid assay (Thermo). Equal amounts of protein (20–30 μg) were loaded on 12% sodium dodecyl sulfate polyacrylamide gels and transferred to polyvinylidene difluoride membranes (Millipore) using wet transfer (100V, 1 h) as described (Hong et al. 2023). Membranes were blocked in 5% Bovine serum albumin in Tris-buffered saline with 0.1% Tween-20 for 1 h at room temperature and washed in phosphate-buffered saline with 0.5% Tween-20. Primary antibody incubations were performed overnight at 4°C in blocking buffer. Antibodies used: anti-PMP2 (ProteinTech, Cat. No. 12717-1-AP, RRID:AB_2166978), anti-GFP (Clontech, Cat. No. 632380, RRID:AB_10013427), anti-Calnexin (Sigma, Cat. No. C4731; loading control, RRID:AB_476845), all at a 1:1000 dilution. Horseradish peroxidase-conjugated secondary antibodies (1:5000, Jackson Labs) were applied for 1 h at room temperature. Detection was performed using Enhanced Chemiluminescence reagents (Bio-Rad) and imaged using a BioRad GelDoc system. Band intensities were quantified with ImageJ software and normalized to calnexin.

### 2.9 | Statistical Analyses

Experiments were not randomized, but data collection and analysis were performed blind to the conditions of the experiments. Data are presented as mean ± standard deviation. No formal a priori sample size calculation was performed for this study. Instead, sample sizes were determined based on established practice in similar in vitro studies examining Schwann cell metabolism and myelination (Catignas et al. 2021; Della-Flora Nunes et al. 2017; Hong et al. 2024; Poitelon et al. 2015; Acheta et al. 2025). No outliers were excluded. Data distribution was assessed before applying parametric statistical tests. Normality was evaluated using the Shapiro–Wilk test. For datasets where *p* > 0.05, normality was assumed, and parametric analyses were used. Two-tailed Student’s *t*-test and one-way analysis of variance (ANOVA) were used for statistical analysis of the differences between multiple groups according to the number of sample groups. Values of *p* ≤ 0.05 were considered to represent a significant difference.

## 3 | Results

### 3.1 | Isolated Schwann Cells at Different Ages Show Similar Metabolic Responses

Schwann cell maturation is known to involve significant changes in morphology, myelination capacity, and gene expression; however, whether their metabolic profiles shift accordingly remains unclear. To determine whether Schwann cells undergo metabolic changes during postnatal development, we compared mitochondrial and glycolytic activity in mouse Schwann cells isolated at early (postnatal Day 5, P5) and more mature (P40) ages. To assess the effect of maturation on Schwann cell respiration, we used a mitochondrial stress test, which provides information on the resting respiration of cells (basal respiration), total respiratory capacity (maximal respiration), the percentage of respiration linked to ATP production, and oxygen consumption due to mitochondrial inner membrane proton leak (Figure 1A). Quantitative analysis revealed no statistically significant differences in basal or maximal respiration, proton leak, or ATP production pathways, suggesting that Schwann cells present a similar metabolic phenotype from early postnatal ages to young adulthood (Figure 1B–H). These findings imply that either in vivo myelination does not substantially impact Schwann cell energy metabolism, or that any transient metabolic changes associated with in vivo myelination are lost during in vitro isolation and culture.

### 3.2 | Overexpression of PMP2 Increases Mitochondrial ATP Production in Isolated Schwann Cells

Given that in other cellular systems, fatty acid-binding proteins (FABPs) are thought to direct fatty acids toward specific intracellular destinations, including mitochondria for β-oxidation and ATP synthesis, it is essential to investigate how PMP2 may impact mitochondrial metabolism in Schwann cells. Our previous work showed that loss of PMP2 reduces mitochondrial ATP production in sciatic nerves during development (Hong et al. 2024). Under NRG1t3-driven hypermyelination, loss of PMP2 expression triggers a metabolic shift toward glycolytic ATP production, which maintains overall ATP levels (Hong et al. 2024). However, following nerve crush, and during NRG1t3-driven hyper-remyelination, glycolysis fails to compensate for the mitochondrial deficit caused by PMP2 loss, which results in reduced basal respiration and total ATP production, associated with the formation of thinner myelin (Hong et al. 2024).

**FIGURE 2 |:**
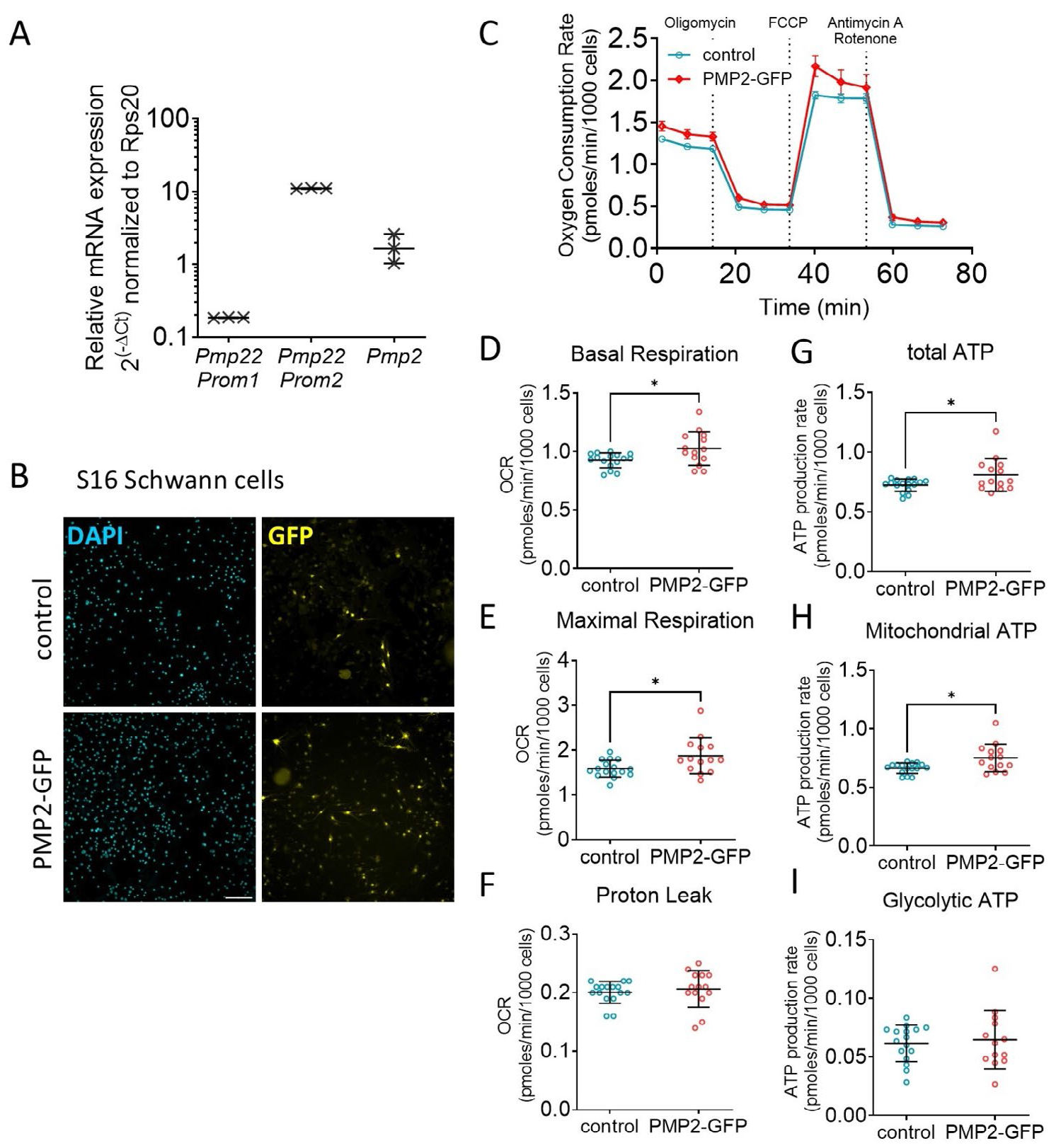
Overexpression of PMP2 modulates S16 Schwann cell metabolism. (A) RTqPCR for *Pmp22* promoter 1, *Pmp22* promoter 2 and *Pmp2* in S16 Schwann cells. *Rsp20* was used as endogenous control. *n* = 3 biological replicates. (B) Immunohistochemistry for DAPI and GFP in S16 Schwann cells infected with GFP (control) or PMP2-GFP. Scale bar = 100 μm (C) Oxygen consumption rate in control (blue-colored labels) and PMP2-GFP (red-colored labels) S16 Schwann cells. (D–I) Basal respiration (D), maximal respiration (E), proton leak (F), total ATP production (G), mitochondrial ATP (H), and glycolytic ATP production (I) were extrapolated from the OCR and ECAR measurements in control and PMP2-GFP S16 Schwann cells. Error bars represent SD, *n* = 16 wells from 3 distinct experiments, and each point on the graph represents a different *n*. Unpaired Student’s *t*-test. *p*_basal respiration_ = 0.0166 (*t* = 2.548, df = 28), *p*_max respiration_ = 0.0161 (*t* = 2.561, df = 28), *p*_total ATP_ = 0.0277 (*t* = 2.322, df = 28), *p*_mito ATP_ = 0.0102 (*t* = 2.756, df = 28). *p value < 0.05.

**FIGURE 3 |:**
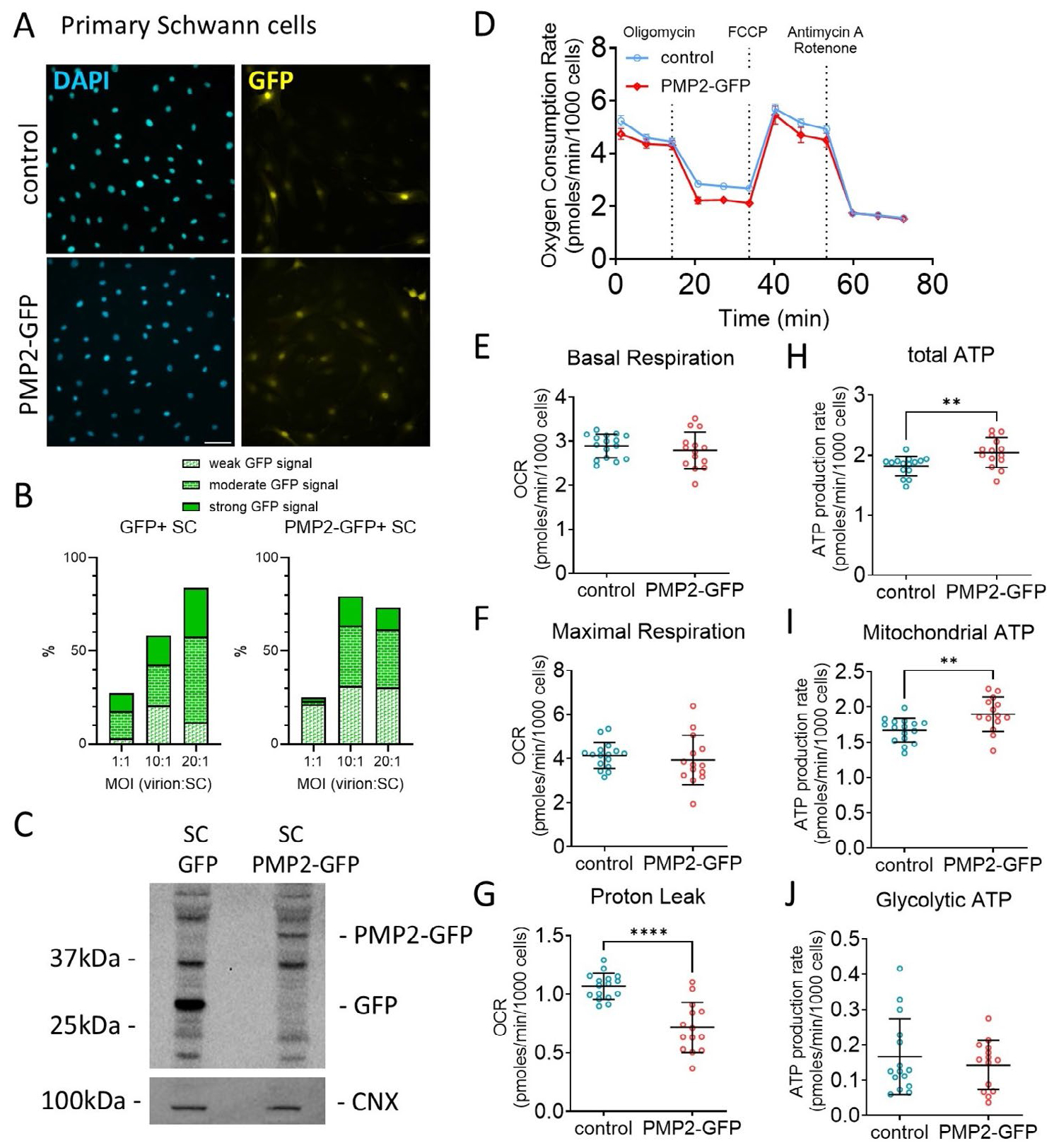
Overexpression of PMP2 modulates primary Schwann cell metabolism. (A) Immunohistochemistry for DAPI and GFP in S16 Schwann cells infected with GFP (control) or PMP2-GFP. Scale bar = 100 μm. (B) Quantification of the number of GFP positive Schwann cells when infected with a multiplicity of infection of 1, 10, and 20 virions per Schwann cells. (C) Western Blot for GFP in rat Schwann cells infected with GFP (control) or PMP2-GFP. Calnexin (CNX) was used as loading control. (D) Oxygen consumption rate in control (blue-colored labels) and PMP2-GFP (red-colored labels) Schwann cells. (E–J) Basal respiration (E), maximal respiration (F), proton leak (G), total ATP production (H), mitochondrial ATP (I), and glycolytic ATP production (J) were extrapolated from the OCR and ECAR measurements in control and PMP2-GFP Schwann cells. Error bars represent SD, *n* = 16 wells from 3 distinct experiments, and each point on the graph represents a different *n*. Unpaired Student’s *t*-test. *p*_proton leak_ < 0.0001 (*t* = 5.762, df = 28), *p*_total ATP_ = 0.0074 (*t* = 2.894, df = 28), *p*_mito ATP_ = 0.0056 (*t* = 2.998, df = 28). ****p value < 0.0001, **p value < 0.01, *p value < 0.05.

Building on our previous finding that axonal neuregulin-mediated modulation of PMP2 expression influences Schwann cell energy homeostasis, we now aim to determine whether PMP2 overexpression alone can affect Schwann cell respiration and ATP production. First, we used the S16 rat Schwann cell immortalized line, which has been shown to sustain high levels of myelin protein expression comparable to those in myelinating Schwann cells and possesses transcriptional regulation similar to rat sciatic nerve (Lopez-Anido et al. 2016; Jones et al. 2011) (Figure 2). We demonstrated that S16 cells express the *Pmp22* transcripts regulated by the Schwann cell-specific *Pmp22* P1 promoter, which drives the majority of *Pmp22* expression in rodents; *Pmp22* transcripts regulated by the P2 promoter, which predominates in other tissues and cultured Schwann cell lines such as S16 (Pantera et al. 2018); and *Pmp2* transcripts (Figure 2A). Second, we used primary rat Schwann cells, which retain the capacity to myelinate neurons in vitro (Poitelon and Feltri 2018) (Figure 3). Both S16 and primary rat Schwann cells were infected with control GFP and PMP2-GFP virions. GFP and PMP2-GFP expression were confirmed by immunocytochemistry (Figures 2B, 3A,B) and Western Blot (Figure 3C). Using a multiplicity of infection of 10 virions for 1 Schwann cell, we obtained an infection rate for PMP2-GFP of ~80% in both S16 Schwann cells (Figure 2B) and primary Schwann cells (Figure 3A,B).

In S16 Schwann cells, we found that overexpression of PMP2 led to an increase in both basal (+11%) and maximal respiration (+18%) (Figure 2C–E), suggesting heightened metabolic activity and energy demand. These were correlated with an increase in both overall (+12%) and mitochondrial ATP (+13%) production in S16 Schwann cells overexpressing PMP2 (Figure 2G,H). Contrastingly, we did not observe any dysregulation of basal and maximal respiration in primary rat Schwann cells (Figure 3D–F). However, we found that an elevated level of PMP2 led to a decrease in proton leak (protons that migrate back into the mitochondrial matrix without producing ATP) in Schwann cells (−33%) (Figure 3G), which could be coupled to a more efficient oxidative phosphorylation and improved ATP production. Indeed, we found that both overall (+12%) and mitochondrial (+14%) ATP production increased in primary Schwann cells overexpressing PMP2 (Figure 3H,I). Together, while ATP increases remain relatively modest, these findings suggest that PMP2 overexpression enhances mitochondrial function in Schwann cells by increasing ATP production, either through heightened respiration in S16 Schwann cells or improved coupling efficiency in primary Schwann cells. This supports a role for PMP2 in promoting metabolic adaptations that may facilitate the energy demands of myelination.

### 3.3 | PMP2 Alters Mitochondrial Function and Substrate Utilization in Schwann Cells

**FIGURE 4 |:**
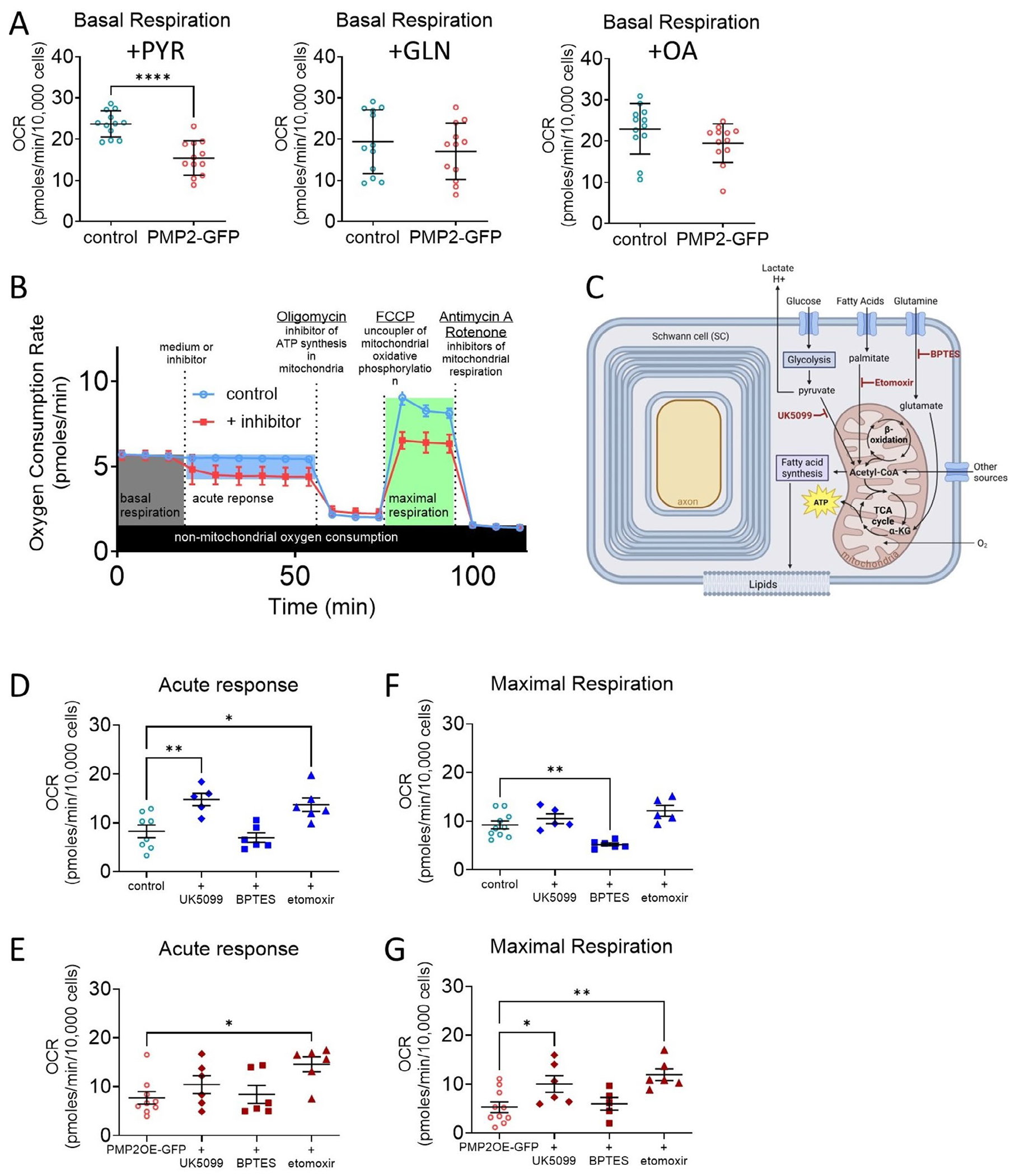
Overexpression of PMP2 modulates Schwann cell metabolic dependencies. (A) Basal respiration in control (blue-colored labels) and PMP2-GFP (red-colored labels) rat Schwann cells. Media was supplemented in Pyruvate, Glutamine, Oleic acid. (B) Schematic representation of the Substrate Oxidation Stress Test. (C) Schematic representation of substrate oxidation inhibitor drugs. Acute response (basal reliance on a substrate) (D, E) and maximal respiration (capacity to utilize a certain substrate when energetic demand is high) (F, G) were extrapolated from the OCR measurements in control and PMP2-GFP Schwann cells treated with UK5099, BPTES, or etomoxir. Error bars represent SD, *n* = 8 wells from 3 distinct experiments, and each point on the graph represents a different *n*. Unpaired Student’s *t* test (A). *p*_+PYR_ < 0.0001 (*t* = 5.475, df = 22). One-Way ANOVA (D-G). *p*_D_ = 0.0005 (*F* = 0.7944 (DFn = 3, DFd = 21)), Bonferroni post hoc comparison: *p*_+UK5099_ = 0.005, *p*_+etomoxir_ = 0.014. *p*_E_ = 0.0267 (*F* = 0.3327 (DFn = 3, DFd = 23)), Bonferroni post hoc comparison: *p*_+etomoxir_ = 0.0136. *p*_F_ = 0.0002 (*F* = 1.259 (DFn = 3, DFd = 22)), Bonferroni post hoc comparison: *p*_+etomoxir_ = 0.0052. *p*_G_ = 0.0035 (*F* = 0.6309 (DFn = 3, DFd = 23)), Bonferroni post hoc comparison: *p*_+UK509_ = 0.0395, *p*_+etomoxir_ = 0.0031. ****p value < 0.0001, **p value < 0.01, *p value < 0.05.

Metabolic dependencies refer to a cell’s reliance on specific substrates or pathways, such as glucose and pyruvate, glutamine, or fatty acids, for energy production, while metabolic capacities describe the cell’s potential to engage those pathways under increased energetic demand. Prior to measuring substrate dependency, we assess basal respiration when the culture media were supplemented with pyruvate, glutamine, or oleic acid (Figure 4A). Among the tested substrates, pyruvate supplementation reduced basal respiration (−35%) in PMP2-overexpressing Schwann cells (Figure 4A), suggesting that, in the presence of exogenous pyruvate, basal respiration is reduced in PMP2-overpressing Schwann cells, possibly reflecting that PMP2 regulates a pyruvate-induced feedback inhibition of mitochondrial ATP production.

To assess how PMP2 overexpression affects Schwann cell metabolic dependency and flexibility, we then performed a substrate oxidation stress assay. This assay distinguishes between baseline reliance on a substrate (acute response) and the Schwann cell’s dependency to utilize a certain substrate when energy demand is high (maximal response) (Figure 4B). To evaluate specific pathways, Schwann cells were treated with substrate oxidation inhibitors: UK5099, an inhibitor of plasma membrane monocarboxylate transporters; BPTES, a glutaminase inhibitor; or etomoxir, an inhibitor of fatty acid oxidation (Figure 4C).

In control Schwann cells, treatment with UK5099 or etomoxir increases acute oxygen consumption (+78% and +65%, respectively), suggesting that following pyruvate or fatty acid oxidation blockage, rapid compensatory responses lead to the oxidation of alternative substrates to support basal mitochondrial activity (Figure 4D). Maximal respiration in control Schwann cells treated with UK5099 or etomoxir remained unchanged, suggesting that, under high energy demand and despite inhibition of pyruvate transport or fatty acid oxidation, the electron transport chain retains its full capacity (Figure 4F). Contrastingly, treatment with BPTES does not affect the acute oxygen consumption rate in control Schwann cells but significantly reduces their maximal oxygen consumption by 43% (Figure 4F), suggesting that glutamine metabolism is important for maintaining Schwann cell mitochondrial function capacity following high energy demand.

In PMP2-overexpressing Schwann cells, treatment with UK5099 did not significantly alter acute oxygen consumption, while maximal respiration increased by 85% (Figure 4E,G), suggesting that low reliance on pyruvate as PMP2-overexpressing Schwann cells can upregulate mitochondrial capacity using alternative substrates under high energy demand. Similarly, following etomoxir treatment, we observed an increase in acute oxygen rate (+88%), coupled to an increase in maximal oxygen consumption (+85%) in PMP2-overexpressing Schwann cells, which also suggests that when fatty acid oxidation is impaired, PMP2 may also promote greater metabolic adaptability and mitochondrial reserve capacity (Figure 4E,G). Finally, in contrast to control Schwann cells, treatment with BPTES did not affect PMP2-overexpressing Schwann cell oxygen consumption (Figure 4G).

Overall, these results indicate that control Schwann cells are not dependent on pyruvate transport or fatty acid oxidation under acute or high metabolic stress. Instead, Schwann cells present high metabolic flexibility under acute stress, as oxygen consumption was increased following inhibition of pyruvate transport or fatty acid oxidation. Also, Schwann cells may rely more on glutamine during high metabolic stress. Finally, overexpression of PMP2 modulates Schwann cell metabolic flexibility and may eliminate the reliance of Schwann cells on glutamine, suggesting metabolic reprogramming could affect Schwann cell energy homeostasis following high metabolic stress.

### 3.4 | Schwann Cells Maintain Metabolic Flexibility Under Dual Substrate Restriction

To determine whether PMP2 overexpression in Schwann cells influences mitochondrial substrate oxidation capacity, we performed a mitochondrial substrate oxidation stress assay under dual substrate restriction conditions. Unlike single-substrate inhibition approaches, this method enables assessment of the cells’ ability to compensate through oxidation of an alternative substrate when two primary fuel sources are blocked. To measure glycolytic capacity, Schwann cells were co-treated with BPTES and etomoxir. To assess glutaminolysis capacity, Schwann cells were co-treated with UK5099 and etomoxir. To measure fatty acid β oxidation capacity, Schwann cells were co-treated with both UK5099 and BPTES.

**FIGURE 5 |:**
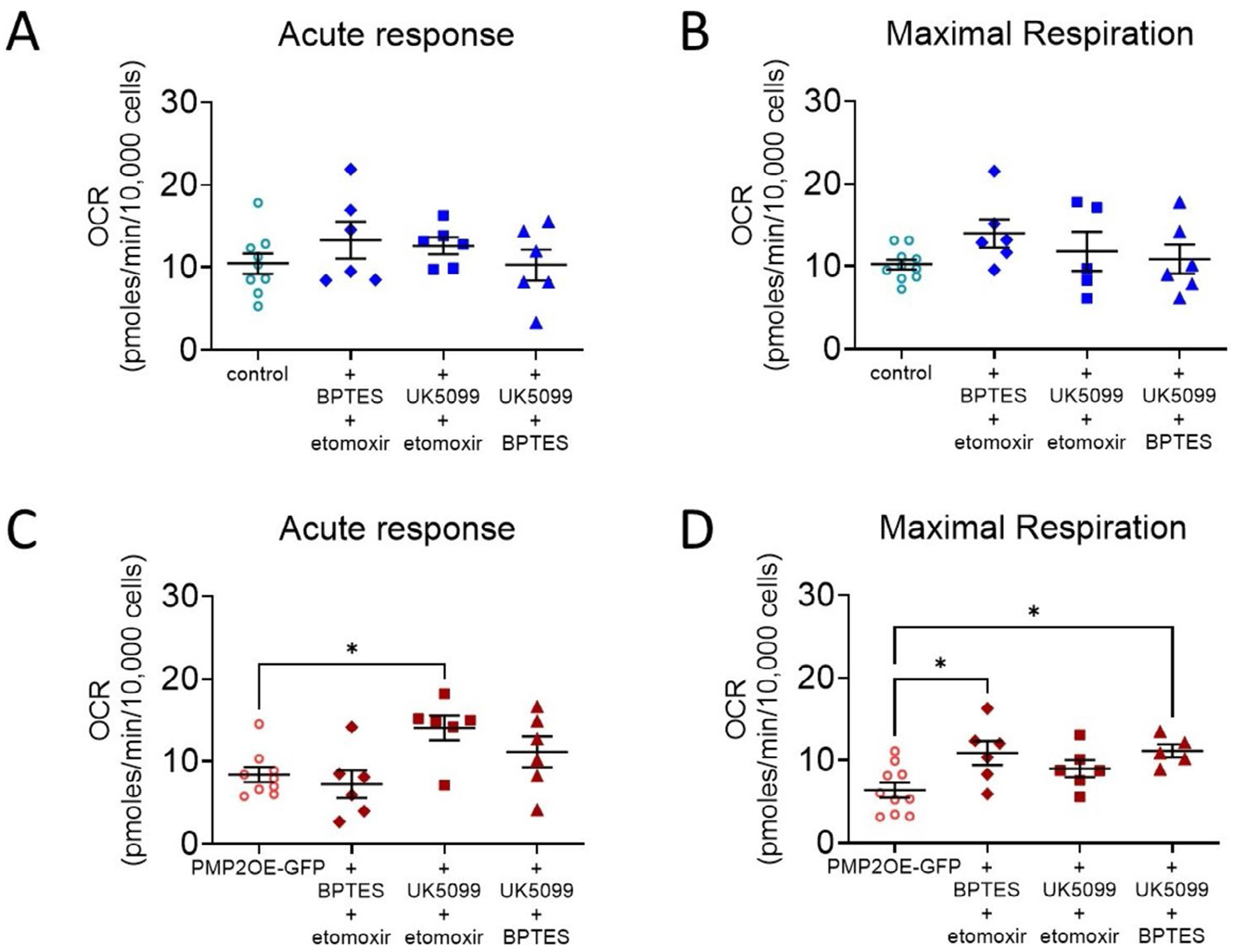
Overexpression of PMP2 modulates Schwann cell metabolic capacities. Acute response (A, C) and maximal respiration (B, D) were extrapolated from the OCR measurements in control (blue-colored labels) and PMP2-GFP (red-colored labels) rat Schwann cells treated with BPTES and etomoxir, UK5099 and etomoxir, and BPTES and UK5099. Error bars represent SD, *n* = 8 wells from 3 distinct experiments, and each point on the graph represents a different *n*. One-Way ANOVA. *p*_C_ = 0.015 (F = 0.800 (DFn = 3, DFd = 23)), Bonferroni post hoc comparison: *P*_+UK5099+etomoxir_ = 0.0234. *p*_D_ = 0.0104 (*F* = 0.7719 (DFn = 3, DFd = 23)), Bonferroni post hoc comparison: *P*_+BPTES+etomoxir_ = 0.015, *p*_+UK5099+BPTES_ = 0.0158. **p value < 0.01, *p value < 0.05.

Surprisingly, and in contrast to results obtained by studying Schwann cell dependencies (Figure 4), none of the co-treatment with substrate inhibitors reduce oxygen consumption in control Schwann cells’ acute response (Figure 5A) or maximal respiration (Figure 5B), suggesting that Schwann cells can compensate for the inhibition of several metabolic pathways by using the only metabolic pathway available.

In PMP2-overexpressing Schwann cells, when glutamine is the only available substrate (following UK5099 + Etomoxir treatment), acute oxygen consumption increases by 82% (Figure 5C), suggesting that PMP2 enhances the capacity of Schwann cells to uptake and utilize glutamine in the absence of other substrates. Similarly, when pyruvate is the only available substrate (following BPTES + Etomoxir treatment) and when fatty acids are the only available substrate (following UK5099 + BPTES treatment), maximal oxygen consumption increases by 106% and by 116%, respectively, in PMP2-overexpressing Schwann cells (Figure 5D). Overall, these results indicate that both control and PMP2-overexpressing Schwann cells exhibit robust metabolic flexibility, with the ability to compensate for substrate restrictions by engaging alternative oxidative pathways.

### 3.5 | Mitochondrial Respiration Is Highly Reduced During Myelination

**FIGURE 6 |:**
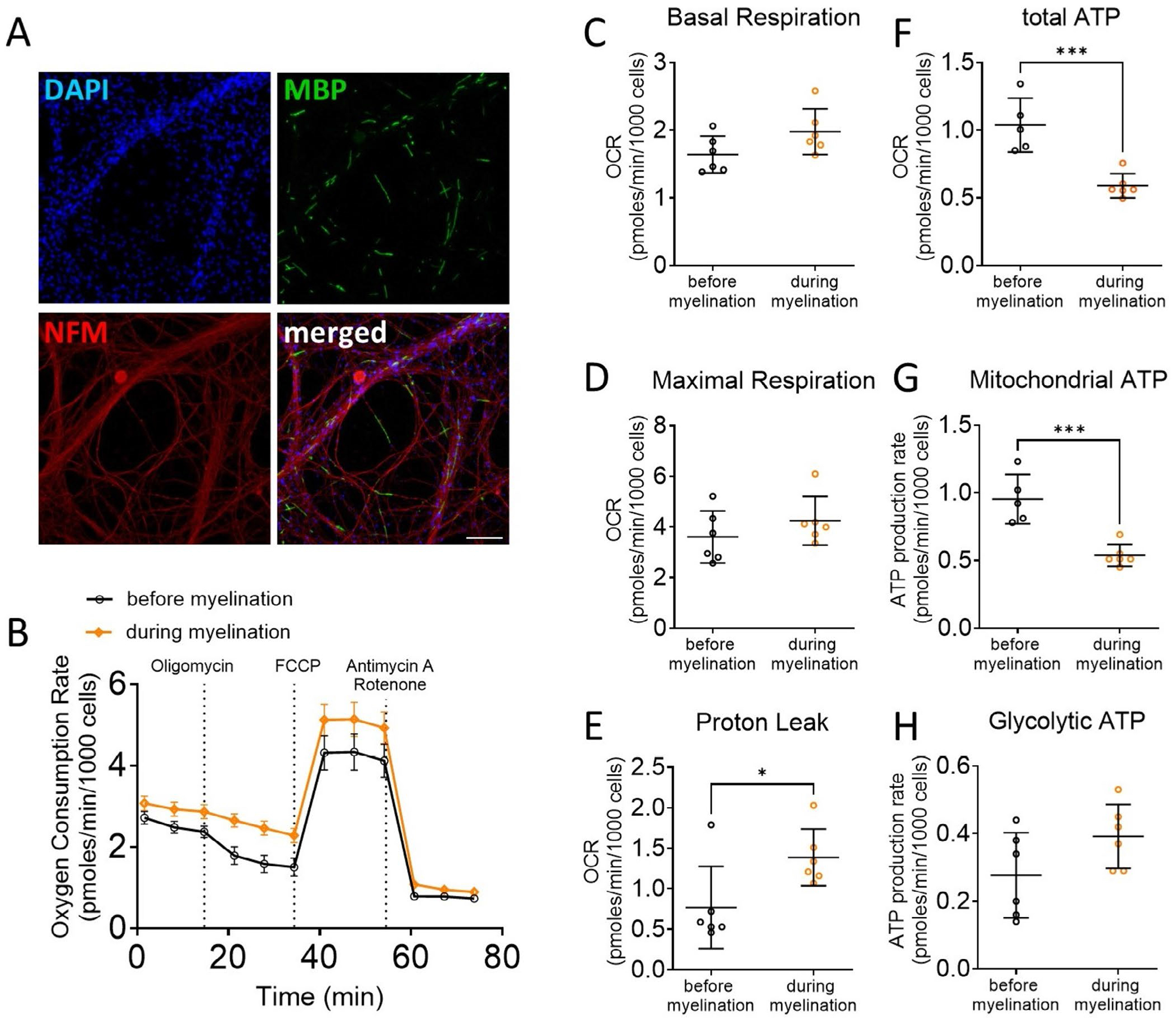
Myelination is coupled with a reduction in ATP production. (A) Immunohistochemistry of rat Schwann cells seeded on top of dorsal root ganglion neurons. Neurons are stained for neurofilament (NFM) in red. Myelin segments are stained for Myelin Basic Protein (MBP) in green. Nuclei are stained with DAPI in blue. (B) Oxygen consumption rate in Schwann cell-neuron coculture before myelination (black-colored labels) and after 10 days of ascorbic acid treatment (orange-colored labels). (C–H) Basal respiration (C), maximal respiration (D), proton leak (E), total ATP production (F), mitochondrial ATP (G) and glycolytic ATP production (H) were extrapolated from the OCR and ECAR measurements in cocultures before and during myelination. Error bars represent SD, *n* = 6 wells, and each point on the graph represents a different *n*. Unpaired Student’s *t*-*test*. *p*_proton leak_ = 0.0343 (*t* = 2.45, df = 9), *p*_total ATP_ = 0.0008 (*t* = 4.985, df = 9), *p*_mito ATP_ = 0.0007 (*t* = 5.05, df = 9). ***p value < 0.001, *p value < 0.05.

Schwann cells undergo significant metabolic remodeling during differentiation and myelination, shifting from a proliferative, energy-demanding state to one optimized for lipid synthesis and support of axonal function. Myelin production is an energetically costly process, relying on extensive lipid biosynthesis, membrane wrapping, and modulation of intracellular signaling (Saher et al. 2005; Montani et al. 2018). While high ATP levels are essential for initiating and sustaining this biosynthetic activity, recent studies suggest that mature myelinating glia may downregulate oxidative phosphorylation once a stable myelin sheath is formed, potentially to minimize reactive oxygen species (ROS) generation and preserve axonal integrity (Viader et al. 2011; Viader et al. 2013; Funfschilling et al. 2012). Furthermore, it has been shown that Schwann cells can support injured axons’ survival through a protective glycolytic shift (Babetto et al. 2020). To evaluate the metabolic changes accompanying Schwann cell myelination, we analyzed oxygen consumption in neuron–Schwann cell cocultures before and after the induction of myelination with ascorbic acid (Figure 6A). While basal respiration and maximal respiration are unchanged (Figure 6C,D), we observe a marked reduction in global (−44%) and mitochondrial ATP production (−44%), along with a significant increase in proton leak (+80%), during myelination (Figure 6E–G). These results suggest that Schwann cells shift away from efficient ATP production, perhaps prioritizing lipid synthesis to support myelination and axonal support.

### 3.6 | PMP2 Overexpression Enhances Schwann Cell Myelination In Vitro

**FIGURE 7 |:**
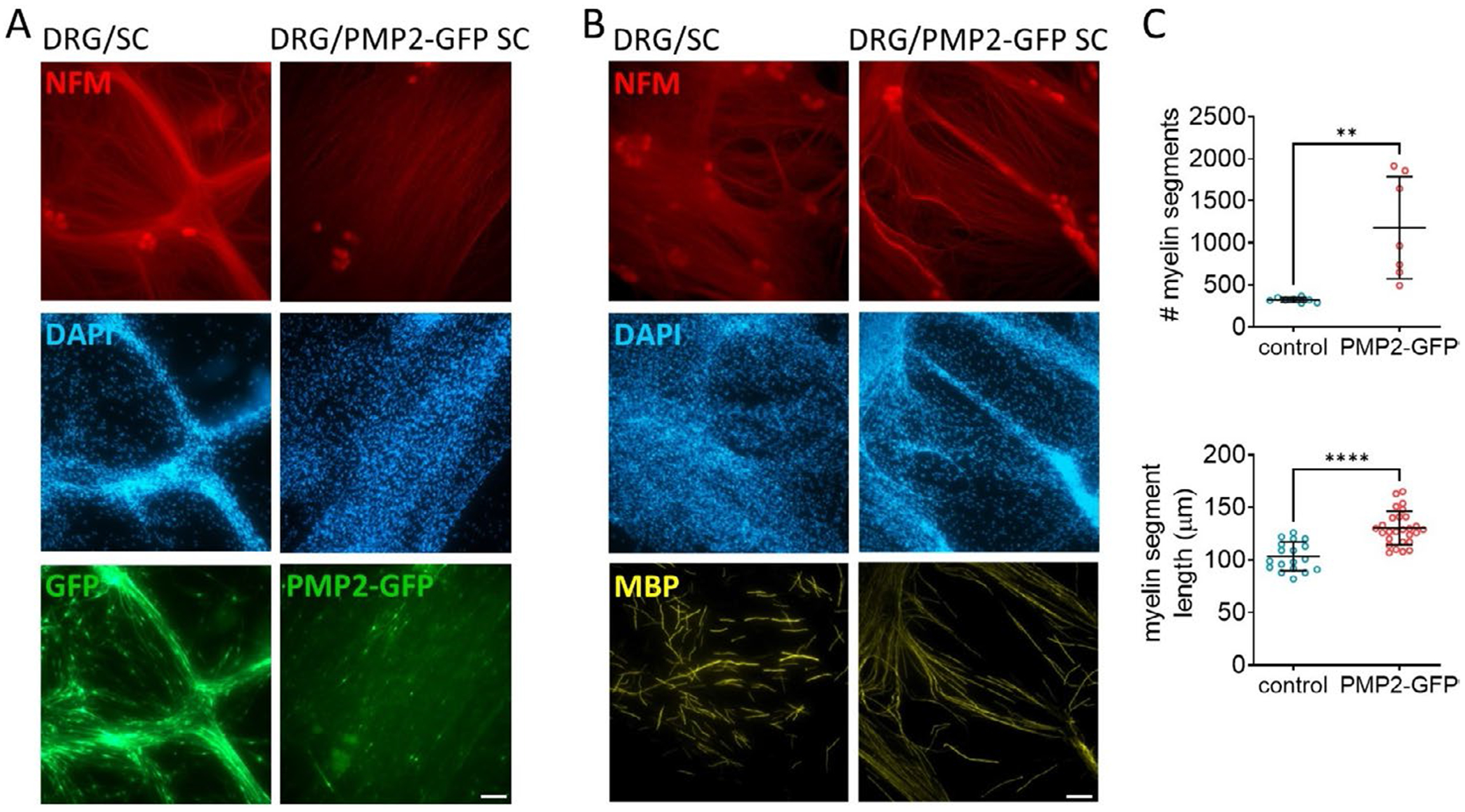
Overexpression of PMP2 increases Schwann cell myelination in vitro. (A) Immunohistochemistry of GFP (control) and PMP2-GFP rat Schwann cells seeded on top of dorsal root ganglion (DRG) neurons. Neurons are stained for neurofilament (NFM) in red. (B, C) Immunohistochemistry (B) and quantification (C) of myelinated segments (MBP, yellow) in cocultures of DRG neurons and control or PMP2-GFP Schwann cells. Scale bar = 50 μm. Error bars represent SD, (B) *n* = 8 coverslips from 3 distinct experiments; (C) *n* = 2 field from 8 coverslips from 3 distinct experiments; each point on the graph represents a different *n*. Unpaired Student’s *t*-test. *p*_myelin segment_ = 0.0015 (*t* = 4.019, df = 13), *p*_segment length_ < 0.0001 (*t* = 5.853, df = 43). ****p value < 0.0001, **p value < 0.01.

In prior studies, we demonstrated that PMP2 is required for NRG1t3-mediated hyper-remyelination and regulates fatty acid uptake in peripheral nerves, suggesting a gain of function role for PMP2 in lipid metabolism (Hong et al. 2024). In the present study, we further implicate PMP2 in energy production, extending its functional role in Schwann cell metabolism. To assess if, independently from Nrg1t3, PMP2 overexpression in Schwann cells increases myelination, we use cocultured wild-type rat DRG neurons with either our GFP control or PMP2-GFP infected rat primary Schwann cells (Figure 7). Immunostaining reveals an increase in the number of myelin basic protein (MBP)-positive segments in cultures by 265% with PMP2-overexpressing Schwann cells compared to controls (Figure 7C). Additionally, a 26% increase in the length of myelinated segments was observed in PMP2-GFP cultures (Figure 7C). These results suggest that modulating PMP2 expression level itself would be sufficient to increase myelination in the peripheral nervous system.

## 4 | Discussion

Our study provides new insights into the metabolic regulation of Schwann cells during development and myelination, with a particular focus on the role of PMP2. We show that, in culture, Schwann cells isolated from early postnatal development to young adulthood maintain a stable metabolic profile under basal conditions. However, overexpression of PMP2 may enhance mitochondrial ATP production through increased respiration or improved oxidative phosphorylation efficiency in Schwann cells. Furthermore, PMP2 overexpression alters substrate utilization patterns and reduces glutamine dependency, indicating metabolic reprogramming. Despite these shifts, Schwann cells retain their metabolic flexibility under multiple substrate restrictions. We demonstrate that myelination is also associated with a decrease in mitochondrial ATP production, suggesting a metabolic shift away from oxidative phosphorylation and/or increased mitochondrial uncoupling, potentially to support anabolic demands or limit oxidative stress during myelin synthesis. Finally, PMP2 overexpression enhances myelination in vitro, suggesting a functional link between Schwann cell metabolism and myelination.

### 4.1 | In Vitro, Schwann Cells Maintain a Stable Metabolic Profile

First, we noted that Schwann cells appear to have a limited oxidative reserve capacity, indicating that they operate near their maximal respiratory rate under basal conditions. This suggests that under basal conditions, Schwann cells are highly metabolically active, in contrast to other metabolically active cells, like isolated muscle cells, which display low basal oxygen consumption and a high reserve capacity (Yang et al. 2017). In addition, our analysis of Schwann cells isolated at postnatal Day 5 (P5) and Day 40 (P40) revealed comparable mitochondrial and glycolytic activity. These results could suggest that Schwann cell core metabolic programming is established early and remains largely unchanged during postnatal development, or that any metabolic reprogramming occurring in vivo is not retained once the SCs are isolated, with metabolic adaptations being lost during in vitro culture. This relative metabolic stability contrasts with other glial lineages, such as oligodendrocytes, where differentiation is tightly coupled to dynamic metabolic transitions. In oligodendrocyte precursor cells (OPCs), maturation into myelinating oligodendrocytes involves a shift from high reliance on oxidative phosphorylation to increased glycolytic activity, as their need for mitochondrial respiration diminishes following the completion of myelination (Meyer and Rinholm 2021; Tepavcevic 2021). This metabolic switch is crucial, as disruptions in mitochondrial function or ATP availability can impair oligodendrocyte differentiation and myelination (Rosko et al. 2019). Together, these findings underscore the central role of metabolic flexibility in supporting oligodendrocyte development and CNS myelination. In contrast, our data support that Schwann cells adopt a relatively fixed metabolic state once isolated in vitro, regardless of developmental age. This observation could support the idea that Schwann cell metabolic plasticity may depend more on extrinsic cues, such as axon-derived signals or injury responses, rather than intrinsic, age-dependent programming. Indeed, unlike oligodendrocytes, Schwann cells require live, juxtacrine signaling from axons to initiate the myelination process (Poitelon et al. 2015). This suggests that Schwann cells may only modulate their metabolic activity, particularly oxidative metabolism, when gaining or losing axonal interaction and during periods of active myelination or regeneration. While this is an intriguing hypothesis, it remains difficult to test with the Clark electrode or Seahorse approach, as measuring extracellular acidification and oxygen consumption in Schwann cell/neurons co-cultures also captures neuronal metabolic activity. Also, we cannot rule out the possibility that oxidative phosphorylation in neurons is altered upon interaction with Schwann cells. Nevertheless, we reveal that Schwann cell myelination may be associated with a reduction in mitochondrial ATP production and a concomitant increase in proton leak. These changes suggest a strategic metabolic downshift once myelination is underway, likely to minimize ROS production and support long-term axonal maintenance via lactate shuttling (Viader et al. 2013; Deck et al. 2022; Babetto et al. 2020; Funfschilling et al. 2012). Thus, the decline in mitochondrial respiration during myelination may represent a conserved mechanism to balance biosynthetic output with metabolic safety in nerve tissue.

### 4.2 | Schwann Cells Are Reliant on Glutamine Metabolism

In our metabolic assays, inhibiting glutaminase with BPTES, a compound that blocks the conversion of glutamine to glutamate, significantly reduced oxygen consumption in primary Schwann cells, highlighting their dependence on this pathway. In addition, we observed that an increase in oxygen consumption upon inhibition of pyruvate and fatty acid pathways in PMP2-overexpressing Schwann cells may reflect the cells’ ability to adapt their glutamine metabolism, potentially by upregulating glutamine metabolism to maintain mitochondrial respiration and ATP production. This metabolic adaptability likely helps Schwann cells preserve energy homeostasis under varying substrate availability, supporting both their basal functions and the biosynthetic demands of myelination and axonal maintenance.

The role of glutamine as an energy substrate by myelinating glial cells has been seldom explored. However, both Schwann cells (Pestoni et al. 2019) and oligodendrocytes (Sykes et al. 1986; Kula et al. 2019; Turan et al. 2021; Braaker et al. 2025) can utilize glutamine for energy production. In addition, the metabolism of glutamine in Schwann cells is dynamic, with an increase in glutaminolysis being observed in the context of schwannoma-associated merlin deficiency (Pestoni et al. 2019). Glutamine can also be converted to glutamate, which can also serve as a signaling molecule influencing Schwann cell differentiation. Activation of glutamate receptor 2 (mGluR2) by glutamate has been shown to promote Schwann cell proliferation and dedifferentiation, processes essential during nerve repair. Conversely, inhibiting mGluR2 signaling encourages myelination, underscoring the role of glutamate in modulating Schwann cell differentiation (Saitoh et al. 2016). In the CNS, oligodendrocytes express high levels of glutamine synthetase, enabling the synthesis of glutamine from glutamate. Selective deletion of this enzyme in oligodendrocytes leads to reduced brain glutamate and glutamine levels and impairs glutamatergic synaptic transmission, although myelination remains unaffected. This suggests that oligodendrocyte-derived glutamine plays a vital role in supporting neuronal communication (Xin et al. 2019). Furthermore, glutamate signaling is integral to oligodendrocyte development. Neuronal activity releases glutamate, which interacts with receptors on oligodendrocyte precursor cells, promoting their maturation into myelinating oligodendrocytes. Disruption of this signaling pathway can impair myelination, highlighting the importance of glutamate in central nervous system development and function (Turan et al. 2021). Collectively, these insights open avenues for research into how glutamine metabolism and signaling may be regulated by axonglia interaction, used in neuronal trophic support, and may be critical for sustaining the biosynthetic demands of myelin production and maintenance.

### 4.3 | PMP2 Promotes Mitochondrial ATP Production and Myelin Production

Our data establishes a new role for PMP2 in enhancing mitochondrial bioenergetics in both primary Schwann cells and the S16 Schwann cell line. These findings build on prior work showing that PMP2 deficiency impairs mitochondrial ATP production (Hong et al. 2024). Notably, the observed increase in mitochondrial ATP remains modest; thus, additional ATP quantitation methods will be required to confirm this effect and explore it further in future studies. Yet, our findings suggest that PMP2 overexpression alters substrate preference and dependency for energy production in Schwann cells. Particularly, PMP2 overexpression reduces oxygen consumption when pyruvate is in excess (Figure 4A), while reducing Schwann cell reliance on glutamine to sustain mitochondrial respiration (Figure 4F,G). Given that mitochondrial ATP production is reduced during myelination (Figure 6), this supports a metabolic shift away from glucose-dependent mitochondrial respiration toward alternative substrates such as glutamine and fatty acids to meet the energetic and biosynthetic demands of myelin production. In this context, the enhanced expression of PMP2 may facilitate this shift by promoting fatty acid uptake and channeling them toward lipid synthesis for myelination (Hong et al. 2024). Our data further support this hypothesis by showing that, independently of NRG1t3 signaling, PMP2 overexpression in Schwann cells increases both the number and length of myelinated segments in DRG co-cultures (Figure 7). Thus, PMP2 may not only support lipid handling but also support the anabolic demands of myelination.

Under acute and high energy demand, while modestly increasing ATP production, PMP2 significantly reshaped how Schwann cells utilize metabolic substrates. We found that etomoxir treatment increases respiration in both control (Figure 4D) and PMP2-overexpressing Schwann cells (Figure 4E). If Schwann cells relied heavily on fatty acids for their metabolic needs, inhibiting CPT1 with etomoxir would lower respiration. Instead, respiration increases, which suggests that blocking fatty acid oxidation may remove a negative regulatory influence or that the cells are shifting to more efficient or readily available substrates (e.g., glucose, glutamine). In addition, when fatty acid oxidation is the only oxidative pathway available, increasing PMP2 expression may enhance lipid-supported mitochondrial respiration (Figure 5D). This indicates that fatty acid oxidation might not be the primary source for basal energy in Schwann cells, but fatty acids can still be used to support mitochondrial respiration under stress, especially when PMP2 expression is increased. Thus, while PMP2 does not increase absolute respiratory capacity, it increases mitochondrial adaptability, allowing Schwann cells to better meet energy demands under metabolic stress. While PMP2 may participate in lipid trafficking that directly supports membrane remodeling and organelle function during periods of high biosynthetic demand, such as myelination, our results do not rule out the possibility that the effect of enhanced PMP2 expression on metabolic function occurs indirectly. For example, this could occur through modulation of endoplasmic reticulum stress or phosphoinositide-mediated calcium dysregulation. The overexpression of myelin proteins or other abundant membrane-associated proteins can induce endoplasmic reticulum stress, which may lead to altered calcium handling and secondary effects on mitochondrial metabolism (Bravo et al. 2011). In addition, PMP2 is known to bind phosphoinositide (PIPs) (Abe et al. 2021), and dysregulation of PIP signaling can modulate intracellular calcium flux, potentially influencing mitochondrial function (Oh 2023). While we did not directly measure endoplasmic reticulum stress markers or calcium levels in this study, a prior study demonstrates that enhanced PMP2 expression under Neuregulin 1 type 3 overexpression is not associated with endoplasmic reticulum stress (Scapin et al. 2019). Additionally, this study shows that PMP2 overexpression does not impair respiration or reduce mitochondrial efficiency; rather, it enhances mitochondrial ATP production and preserves or improves oxidative coupling. This argues against a maladaptive stress response. Although PMP2 has been shown to bind phosphatidylinositol 4,5-bisphosphate (PIP_2_), to our knowledge, it remains unknown whether this interaction disrupts calcium homeostasis in Schwann cells; further investigations quantifying intracellular calcium dynamics will be necessary. Those questions highlight the need for future work to dissect the interplay between PMP2, PIP signaling, and intracellular calcium dynamics in the context of Schwann cell metabolism.

A last possible confounding element in our results is the independent effect of ascorbate on Schwann cells and neurons. In the context of in vitro myelination, the promyelinating effect of ascorbate is mechanistically dependent on the presence of axons and is not observed in Schwann cells or neurons cultured alone. Based on this established biology, and consistent with previous studies using this co-culture system, we interpreted the ascorbate response as reflecting *bona fide* myelin formation rather than a nonspecific stress or differentiation response. Therefore, we did not include ascorbate-only controls in single-cell cultures, as they are not expected to form myelin-like structures or confound the interpretation of PMP2’s effects on myelination. Nonetheless, ascorbate may have more nuanced metabolic effects on Schwann cells or neurons in isolation. For example, intracellular ascorbate has been shown to inhibit glucose uptake by neurons, favoring lactate utilization instead (Castro, Pozo et al. 2007; Castro et al. 2008). This shift could, in principle, affect neuronal mitochondrial metabolism. However, in our system, we did not observe a corresponding decrease in glycolytic ATP, which argues against a major contribution of this mechanism. Similarly, in Schwann cells, ascorbate treatment does not appear to markedly alter mitochondrial metabolism (Huff et al. 2021). The transcriptomic dataset from that study (GSE137139) also provides an opportunity for further exploration of ascorbate-induced changes in metabolic gene expression. While additional experiments on ascorbate-treated Schwann cells or neurons alone could further strengthen this interpretation, ideally confirming that ascorbate does not alter mitochondrial metabolism in either cell type independently, we believe that the most parsimonious explanation for the metabolic effects observed in our study remains the induction of myelination.

### 4.4 | Summary

Together, these findings position PMP2 as a critical regulator of Schwann cell bioenergetics and myelination. Moving forward, it will be important to elucidate the precise lipid-binding and transport mechanisms by which PMP2 modulates mitochondrial metabolism and to investigate how these effects integrate with axonal signals during development and repair. In vivo studies examining PMP2 overexpression during nerve regeneration or in models of peripheral neuropathy will be essential to determine whether PMP2-enhanced metabolism can be harnessed therapeutically to promote remyelination and functional recovery.

## Data Availability

The data that support the findings of this study are available from the corresponding author upon reasonable request.

## Abbreviations:

ATP: adenosine triphosphate
BPTES: bis-2-(5-phenylacetamido-1,3,4-thiadiazol-2-yl)ethyl sulfide
CNS: central nervous system
DRG: dorsal root ganglion
ECAR: extracellular acidification rate
FCCP: carbonyl cyanide-p-trifluoromethoxyphenylhydrazone
MBP: myelin basic protein
NFM: neurofilament M protein
OCR: oxygen consumption rate
PNS: peripheral nervous system
ROS: reactive oxygen species
RRID: Research Resource Identifier (see scicrunch.org)
